# Effects, costs and feasibility of the ‘Stay Active at Home’ Reablement training programme for home care professionals: study protocol of a cluster randomised controlled trial

**DOI:** 10.1186/s12877-018-0968-z

**Published:** 2018-11-13

**Authors:** Silke F. Metzelthin, Teuni H. Rooijackers, Gertrud A. R. Zijlstra, Erik van Rossum, Marja Y. Veenstra, Annemarie Koster, Silvia M. A. A. Evers, Gerard J. P. van Breukelen, Gertrudis I. J. M. Kempen

**Affiliations:** 1 Department of Health Services Research, Care and Public Health Research Institute (CAPHRI), Faculty of Health, Medicine and Life Sciences, Maastricht University, P.O. Box 616, 6200 MD Maastricht, The Netherlands; 2 Research Centre for Community Care, Faculty of Health, Zuyd University of Applied Sciences, P.O. Box 550, 6400 AN Heerlen, The Netherlands; 3 Burgerkracht Limburg, P.O. Box 5185, 6130 PD Sittard, The Netherlands; 4 Department of Social Medicine, Care and Public Health Research Institute (CAPHRI), Faculty of Health, Medicine and Life Sciences, Maastricht University, P.O. Box 616, 6200 MD Maastricht, The Netherlands; 5 Department of Methodology and Statistics, Care and Public Health Research Institute (CAPHRI), Faculty of Health, Medicine and Life Sciences, Maastricht University, P.O. Box 616, 6200 MD Maastricht, The Netherlands

**Keywords:** Reablement, Ageing in place, Sedentary behaviour, Physical activity, Aged people, Home care, Activities of daily living, Nursing, Behavioural intervention, Prevention

## Abstract

**Background:**

According to the principles of Reablement, home care services are meant to be goal-oriented, holistic and person-centred taking into account the capabilities and opportunities of older adults. However, home care services traditionally focus on doing things *for* older adults rather than *with* them. To implement Reablement in practice, the ‘Stay Active at Home’ programme was developed. It is assumed that the programme leads to a reduction in sedentary behaviour in older adults and consequently more cost-effective outcomes in terms of their health and wellbeing. However, this has yet to be proven.

**Methods/ design:**

A two-group cluster randomised controlled trial with 12 months follow-up will be conducted. Ten nursing teams will be selected, pre-stratified on working area and randomised into an intervention group (‘Stay Active at Home’) or control group (no training). All nurses of the participating teams are eligible to participate in the study. Older adults and, if applicable, their domestic support workers (DSWs) will be allocated to the intervention or control group as well, based on the allocation of the nursing team. Older adults are eligible to participate, if they: 1) receive homecare services by the selected teams; and 2) are 65 years or older. Older adults will be excluded if they: 1) are terminally ill or bedbound; 2) have serious cognitive or psychological problems; or 3) are unable to communicate in Dutch. DSWs are eligible to participate if they provide services to clients who fulfil the eligibility criteria for older adults. The study consists of an effect evaluation (primary outcome: sedentary behaviour in older adults), an economic evaluation and a process evaluation. Data for the effect and economic evaluation will be collected at baseline and 6 and/or 12 months after baseline using performance-based and self-reported measures. In addition, data from client records will be extracted. A mixed-methods design will be applied for the process evaluation, collecting data of older adults and professionals throughout the study period.

**Discussion:**

This study will result in evidence about the effectiveness, cost-effectiveness and feasibility of the ‘Stay Active at Home’ programme.

**Trial registration:**

ClinicalTrials.gov: NCT03293303, registered on 20 September 2017.

## Background

Western countries with ageing populations, such as the Netherlands, have to deal with an increasing demand for health care, while financial resources and manpower are shrinking [1]. One strategy to face this challenge is to enable ‘ageing in place’, which is a common policy in these countries. Consequently, the proportion of older adults in Dutch long-term care facilities is decreasing and home care is becoming more important [2]. This is in line with the preference of most older adults, who want to stay at home for as long as possible, even if they suffer from fragile health and are faced with challenging social situations [3]. However, to enable ‘ageing in place’ it is important that older adults maintain their self-care capabilities.

Previous research has shown that physical activity can positively affect daily functioning of older adults [4–7]. Nevertheless, many community-dwelling older adults have a highly sedentary lifestyle [8]. In general, older adults spend approximately 80% of their awake time in sedentary activities which represents 8 to 12 h per day [9, 10]. Most research on stimulating physical activity of older adults, focuses directly on the behaviour of older adults, for instance by offering an exercise intervention, in group or individual format [7]. However, persuading older adults to become and continue to be physically active is a challenging task. Reasons for this may be a lack of motivation, fear (of falling), depression or a poor understanding of the long-term benefits of physical activity in older adults [11]. An alternative for these (classical) exercise programmes is to integrate physical activity in daily care, for example, in home care.

In the Netherlands, 20% of older adults use home care services [12]. Nurses and domestic support workers (DSWs) support them with personal care (e.g. washing and dressing) or domestic tasks (e.g. cleaning or doing the laundry), respectively. Unfortunately, they mainly provide support by taking over tasks instead of stimulating older adults to be active in physical and daily activities, as they are used to doing things *for* older adults rather than *with* them. This can result in a downward spiral, as they deprive older adults of their opportunities to engage in a routine range of movements necessary for maintaining underlying capabilities, resulting in further deconditioning and functional decline [13–15]. These negative consequences may be prevented by implementing Reablement in home care.

During the last decade, Reablement has been introduced in several countries (i.e. US, UK, New Zealand, Australia, Norway, and Sweden), but there is no internationally accepted definition of Reablement, and consequently a great variation between and even within countries exists in how Reablement is implemented [16, 17]. Nevertheless, Reablement initiatives have in common that day-to-day services are meant to be goal-oriented, holistic and person-centred taking into account the capabilities and opportunities of older adults instead of focusing on disease and dependency [18]. So far, evidence concerning the effectiveness and cost-effectiveness of Reablement is scarce and inconsistent [16, 17, 19–21]. A few studies have shown beneficial results with regard to physical activity [22], daily functioning [23–26], health-related quality of life [27, 28] or health care utilisation/ costs [23, 27–31]. Furthermore, little is known about how Reablement is implemented in practice and which client groups are more likely to benefit from Reablement than others [16]. Consequently, more research in the field of Reablement is needed.

In the Netherlands, recently, the ‘Stay Active at Home’ programme was developed based on international evidence and in close collaboration with Dutch and foreign stakeholders [18]. It is a training programme for home care professionals that aims to provide them with knowledge, self-efficacy, skills and social support to implement Reablement in practice. The feasibility of the programme and the research design have been evaluated in an exploratory trial (ClinicalTrials.gov: NCT02904889 (Smeets RGM, Kempen GIJM, Hansen WAG, Zijlstra GAR, van Rossum E, de Man-van Ginkel JM, et al. Experiences of home care professionals with the Stay Active at Home programme targeting reablement of community-living older adults. A qualitative study, submitted for publication)), which is part of the Basic Care Revisited project [32]. Semi-structured interviews that were conducted with home care professionals during the exploratory trial showed that professionals experienced the ‘Stay Active at Home’ programme as an empowering way to apply Reablement in home care (Smeets RGM, Kempen GIJM, Hansen WAG, Zijlstra GAR, van Rossum E, de Man-van Ginkel JM, et al. Experiences of home care professionals with the Stay Active at Home programme targeting reablement of community-living older adults. A qualitative study, submitted for publication). However, the effectiveness, cost-effectiveness and feasibility of the ‘Stay Active at Home’ programme are not yet known. Therefore, a two-group cluster randomised controlled trial will be conducted to evaluate whether its implementation leads towards a reduction in sedentary behaviour in older adults and thereby an increase in their level of physical activity. Furthermore, we will investigate whether the programme leads to more cost-effective outcomes in terms of older adults’ health and wellbeing. In addition, an extensive process evaluation will be conducted alongside the trial to provide information about 1) the implementation of the ‘Stay Active at Home’ programme; 2) its mechanisms of impact; and 3) contextual factors that may affect implementation and outcomes. This paper describes the study protocol of the cluster randomised controlled trial taking into account the SPIRIT 2013 Statement [33, 34].

## Methods/ design

### Objectives

This study evaluates the ‘Stay Active at Home’ programme. More specifically the aims are to get insight into the programme’s:

1. Effectiveness with regard to sedentary behaviour of older adults (primary outcome). Furthermore, several secondary outcomes will be evaluated: physical activity, daily, physical and psychological functioning and falls *(effect evaluation)*.
2. Cost-effectiveness from a societal perspective *(economic evaluation)*.
3. Feasibility with regard to implementation, mechanisms of impact and contextual factors that may affect its implementation and outcomes *(process evaluation)*.

### Design

A two-group cluster randomised controlled trial will be conducted in the south of the Netherlands. Home care professionals (i.e. nurses and DSWs) in the intervention group will receive the ‘Stay Active at Home’ programme. Professionals in the control group will receive no additional training. Data for the effect and economic evaluation will be collected at client level by performance-based and self-reported measures. In addition, data from client records will be extracted. Data are assessed at baseline and 6 and/or 12 months after baseline. A mixed-methods design will be applied for the process evaluation at the client and professional level. Data will be collected throughout the whole study period. For practical reasons, the recruitment of older adults, the implementation of the programme and the data collection will be conducted in four phases. The recruitment of participants will be conducted between September 2017 and January 2018 (two teams, intervention group), November 2017 and January 2018 (two teams, control group), January and April 2018 (three teams, intervention group) and March and June 2018 (three teams, control group). The trial is registered at www.clinicaltrials.gov (#NCT03293303).

### Setting

This study will be conducted at MeanderGroep South-Limburg (www.meandergroep.com), a large healthcare provider that offers, among other services, different types of home care services in the region of South-Limburg: domestic services (e.g. cleaning and other household chores), personal care (e.g. assistance with bathing or dressing) and nursing services (e.g. wound care and injections). MeanderGroep has divided its region into 7 working areas, which are further subdivided into small-scale self-directed nursing teams, with on average 11 nursing teams per working area (range 3–28). Each team is guided by a district nurse (baccalaureate-educated registered nurse). The other team members are vocationally-trained registered nurses or certified nurse assistants. Domestic support is provided by DSWs, who work individually under supervision of a manager. They are linked to a working area, but not to a specific nursing team.

### Randomisation

**Fig. 1:**
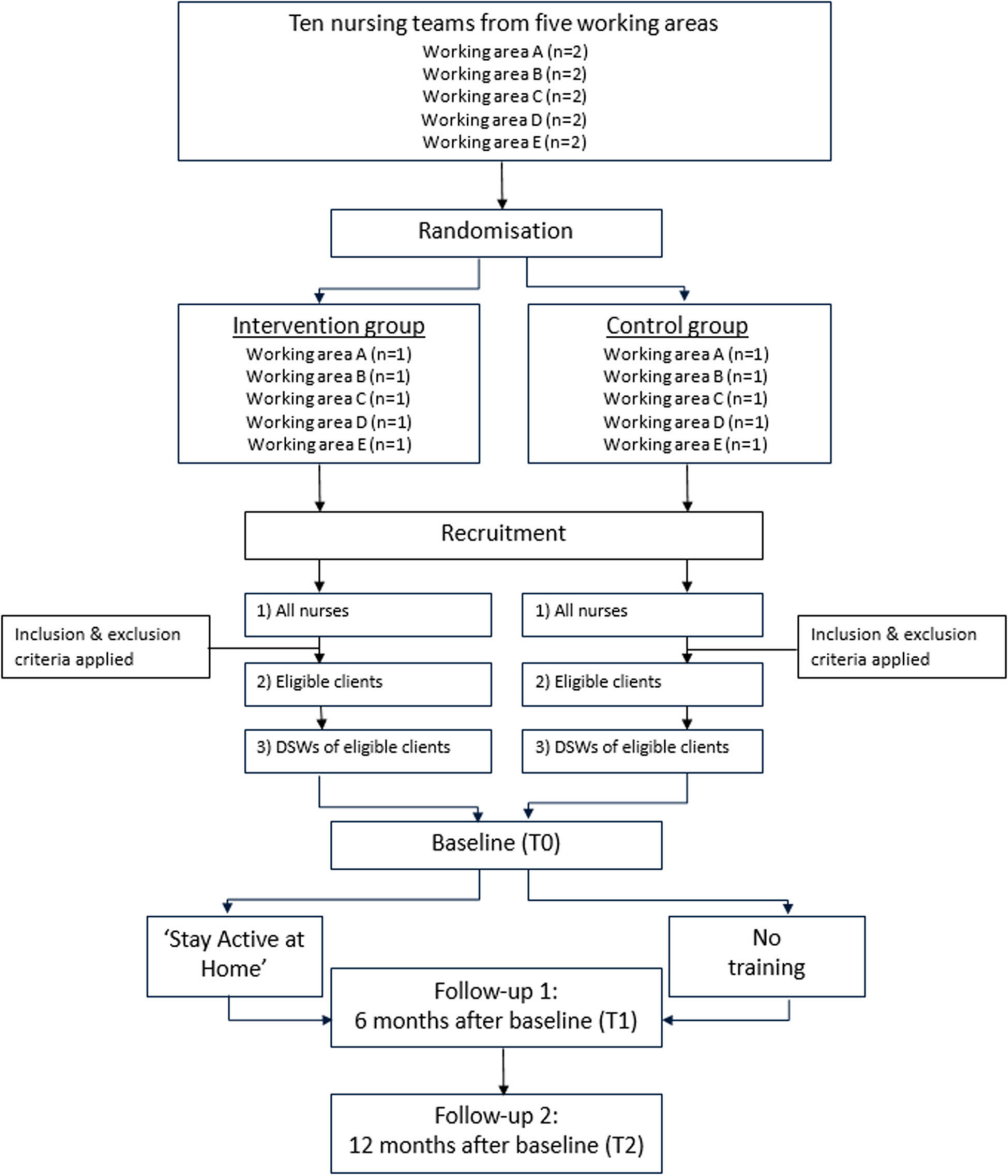
Flow chart of the study

For this study, ten nursing teams in five working areas (two teams per area) will be selected by the nursing team managers of MeanderGroep. To avoid contamination bias, managers will be asked to select two teams within each area that are not collaborating with each other. Furthermore, dementia teams will not be considered, as most of their clients potentially will not fulfil the inclusion criteria for older adults. The nursing teams will be pre-stratified with regard to their working area and randomised into either the intervention or control group within each working area. The clients and, if applicable, their DSWs will be allocated to the intervention or control group based on the allocation of the nursing team. The randomisation will be conducted by means of a computer-generated randomisation list. The researcher who will conduct the randomisation, will be blinded, will not be involved in this study and not familiar with the nursing teams. A flow diagram of the study design is shown in Fig. 1.

### Participants and recruitment

Two types of participants will be recruited for this study: older adults and home care professionals (i.e. nurses and DSWs).

#### Older adults

Several inclusion and exclusion criteria will be determined for older adults. Older adults are eligible to participate in this study if they: 1) receive homecare services by the selected teams; and 2) are 65 years or older. Older adults will be excluded if they: 1) are terminally ill or bedbound; 2) have serious cognitive or psychological problems; or 3) are unable to communicate in Dutch. The participating district nurses, who are leading the nursing teams and are familiar with all clients, will check clients on the eligibility criteria based on their clinical judgement. This will result in a list of eligible clients per team. Subsequently, the recruitment of older adults will start, which consists of three steps. First, older adults will receive a short information letter and flyer about the study on behalf of MeanderGroep. Second, older adults will receive a short telephone call to assess whether they are potentially interested in participating in this study. Third, a home visit will be conducted by the research team (author THR or research assistant) to provide additional information. When older adults agree to participate, the baseline data will be assessed. Participation of older adults is voluntary; they are informed about the study and were asked for written informed consent. Older adults may withdraw from the study for any reason at any time.

#### Home care professionals

All nurses of the participating nursing teams are eligible to participate in the study. There will be no specific inclusion and exclusion criteria for them. DSWs are eligible to participate if they provide services to clients who fulfil the eligibility criteria for older adults. DSWs will be traced via their managers, who receive the list of eligible older adults from the research team (author THR). Based on this list, the manager will inform the research team if clients also receive domestic support of MeanderGroep and by whom. Consequently, these DSWs will be invited to participate in the study.

### Interventions

**Fig. 2:**
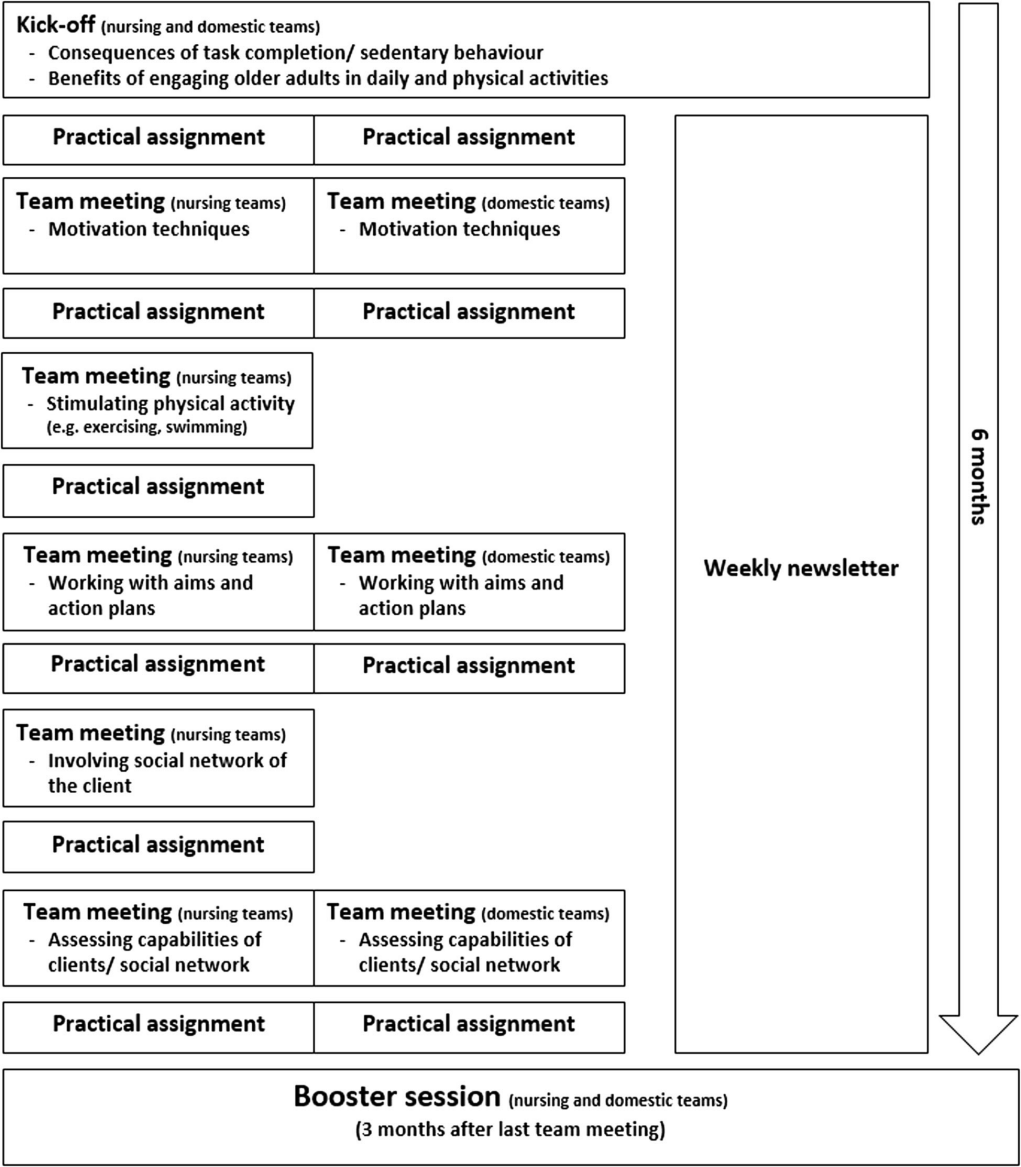
Format and content of the ‘Stay Active at Home’ programme

Home care professionals in the intervention group will follow the ‘Stay Active at Home’ programme. The programme lasts for 9 months and consists of face-to-face meetings, practical assignments in-between the meetings and twenty weekly newsletters. The face-to-face meetings can be subdivided into a kick-off meeting (120 min), a series of (bi-)monthly team meetings (60 min each) which are spread over a period of 6 months, and a booster session (120 min) 3 months later (see Fig. 2 for an overview of the training programme). The kick-off meeting and booster session are the same for nursing teams and DSWs. Professionals from both disciplines, who are working in the same working area, are invited to the sessions to get to know each other. The team meetings are offered to nursing teams and DSWs separately, as these meetings are more focused on discipline-specific tasks. DSWs have fewer team meetings than nursing teams (three and five meetings, respectively), as they have a lower annual time-budget for training activities. During the programme, professionals receive background information about the benefits of Reablement. Furthermore, they learn skills to apply it in practice: (1) assessing capabilities of older adults; (2) implementing goal-setting and action planning; (3) increasing engagement of older adults in physical and daily activities; (4) motivating older adults by taking into account their phase of behavioural change [16, 17, 35, 36] and making use of Bandura’s self-efficacy theory [37, 38]; and (5) involving the social network of older adults. Professionals can practice these skills in a safe environment during the face-to-face meetings. Afterwards they are expected to apply the skills in practice as part of the practical assignments. Their experiences are discussed during the next meeting. Further details about the development and content of the programme are published elsewhere [18]. In addition, a brief movie about the ‘Stay Active at Home’ programme (in Dutch and English) can be found at: https://www.academischewerkplaatsouderenzorg.nl/research-programme/15521.

Professionals in the control group receive no additional training.

### Effect evaluation

Data for the effect evaluation will be collected from older adults using a combination of performance-based and self-reported measures. An overview of all data that will be collected is provided in Table 1. At baseline, data about the primary and secondary outcomes will be assessed during a home visit. Moreover, relevant sociodemographic data of older adults will be collected (i.e. age, gender, educational level, marital status, ethnicity, socio-economic situation and living situation). Another home visit will be conducted 12 months after baseline. Due to a risk of recall bias, data about falls will be collected at both 6 and 12 months after baseline. The data collection at 6 months will be done during a telephone interview, which is primarily conducted to assess data about the economic and process evaluation. All data will be collected by members of the research team (author THR or one of four research assistants). To increase and standardise the quality of data collection, the research team will follow an extended protocol. Author THR will train the research assistants in collecting the data according to this protocol and will be present at their first home visits. Additionally, author THR will monitor the data collection throughout the field work.

**Table 1:** Overview of effect evaluation data collection

Outcomes	Measures	No. of items	Range^a^	Time points		
				*Baseline (T0)*	*Follow-up 1: 6 months after baseline (T1)*	*Follow-up 2: 12 months after baseline (T2)*
Primary outcome measure						
*Sedentary behaviour*	ActiGraph GT9X Link	N/A	N/A	X		X
Secondary outcome measures						
*Self-reported sedentary behaviour*	LASA Sedentary Behaviour questionnaire [46]	10	0–1440 min	X		X
*Physical functioning*	Short Physical Performance Battery [47]	3	0–12	X		X
*Daily functioning*	Groningen Activity Restriction Scale [50]	18	18–72	X		X
*Psychological functioning*	Patient Health Questionnaire-9 [74]	9	9–28	X		X
*Falls*	N/A	1	N/A	X	X	X

^a^underlined scores indicate the most favourable scores; *N/A* not applicable

#### Primary outcome measure

The aim of the ‘Stay Active at Home’ programme is to reduce sedentary behaviour in older adults and thereby increase their level of physical activity. The primary outcome sedentary behaviour at 12 months will be measured by means of a tri-axial wrist-worn accelerometer (ActiGraph GT9X Link, ActiGraph Inc., Pensacola, FL, USA). Accelerometers are a valid and reliable method, also in older adults, to measure sedentary time and physical activity by assessing the magnitude of the body’s acceleration in terms of ‘counts’ per unit time [39–43]. The ActiGraph will be placed on the non-dominant wrist and will be worn for seven consecutive days (24 h per day) at baseline and 12 months after baseline. As the accelerometer will also be worn during the night, information about sleep will also be obtained. Older adults are asked to keep a diary to register when they went to bed, when they got up and when they were napping during the day. Data will be collected at 30 Hz and will be aggregated to 60-s epochs for the analyses [44]. Older adults are required to have at least 1 valid day of 10 h of wake wear time to be included in the analyses. In additional analyses, older adults with four or more valid days will be selected. Waking time and wear time will be defined by an algorithm available in the ActiLife software version 6. Activity counts will be converted into average daily minutes of sedentary behaviour during waking time using a vector magnitude cut-point of < 1853 cpm [45]. In addition, mean wake time activity counts per minute will be compared between groups (secondary outcome).

#### Secondary outcome measures

The LASA Sedentary Behaviour questionnaire [46] will be used to assess self-reported sedentary behaviour. The questionnaire consists of 10 items about sedentary activities such as watching television. Older adults will report the time that they generally spent on each sedentary activity per day. The items must be completed for one weekday and one weekend day. Total self-reported sedentary time (in minutes) for an average day will be calculated as ((total sedentary time on weekdays * 5) + (total sedentary time on weekend days * 2))/7. A previous study has shown that self-reported sedentary time measured by the LASA Sedentary Behaviour questionnaire can reliably rank sedentary time in in older persons and was moderately associated with accelerometer-derived sedentary time [46].

Physical functioning will be measured by the Short Physical Performance Battery (SPPB, [47]). The SPPB is based on three objective tests of physical function: 3-m walking speed, repeated chair stands (five times), and standing balance in progressively more-challenging positions (i.e. feet in side-by-side, semi-tandem, and full-tandem positions). Each test is scored 0 to 4 by previously determined criteria [48]. Scores from the three tests will be summed into a composite score ranging from 0 to 12, with higher scores reflecting better physical functioning. The SPPB has excellent reliability [49] and is highly sensitive to important changes such as self-reported decline in ability to walk a block or to climb one flight of stairs. Decreased SPPB is a strong predictor of nursing home admission, disability in self-care tasks, and mobility in older adults [48, 49].

The Groningen Activity Restriction Scale (GARS, [50]) will be used to collect data about daily functioning. The GARS consists of two subscales and assesses disability in the domains of activities of daily living (ADL, 11 items, such as dressing or getting around in the house) and instrumental activities of daily living (IADL, 7 items, such as preparing breakfast or doing household activities) [50]. For each item, four hierarchical answer options are available ranging from *‘Yes, I can do it fully independently without any difficulty’* to *‘No, I cannot do it fully independently. I can only do it with someone’s help’*. The scores for the total scale range from 18 to 72 with higher scores indicating more disability [50]. The GARS is a reliable and valid measure for assessing disability in the domains of ADL and IADL in older adults [50].

The Patient Health Questionnaire-9 (PHQ-9, [47]) will be used to assess psychological functioning. The PHQ-9 consists of nine items which measure the presence of depressive symptoms according to the Diagnostic and Statistical Manual of Mental Disorders, 4th Edition (DSM-IV). Older adults will score how often each of the symptoms (such as ‘little interest or pleasure in doing things’ or ‘feeling tired or having little energy’) was present during the last two weeks (0 = not at all; 1 = several days; 2 = more than half of the days; 3 = nearly every day). The summary score ranges from 0 to 27, with higher scores reflecting more severe symptoms of depression. The PHQ-9 has been shown to be a valid and reliable instrument to measure depression in community-dwelling older adults [51].

Finally, the frequency of falls will be assessed by the question: *‘How often did you fall during the past 6 months’* [52]. This question is included to monitor a potential negative outcome of physical activity, despite research showed that stimulating older adults to be more active does not necessarily lead to an increase in fall incidents [53].

### Economic evaluation

The economic evaluation will be conducted according to the Dutch guidelines of economic evaluations in health care [54, 55], which were developed in agreement with international standards. A combination of cost-effectiveness analysis (CEA) and cost-utility analysis (CUA) will be performed from a societal perspective, which implies that all relevant outcomes will be taken into account (i.e. intervention costs, health care costs, patient and family costs) [54, 55]. Self-reported data will be collected together with the data for the effect evaluation by the research team at baseline and 6 and/or 12 months after baseline. In addition, data from client records will be extracted at the end of the study. An overview of all collected data is provided in Table 2. The time horizon will be the same period as the follow-up period of the effect evaluation (i.e. 12 months).

**Table 2:** Overview of economic evaluation data collection

Outcomes	Measures	No. of items	Range^a^	Time points		
				*Baseline (T0)*	*Follow-up 1: 6 months after baseline (T1)*	*Follow-up 2: 12 months after baseline (T2)*
Clinical outcomes						
*Sedentary behaviour*	ActiGraph GT9X Link	N/A	N/A	X		X
*Health-related quality of life*	QALYs (based on EuroQol-5D-5 L [56, 57]	5	0–1	X	X	X
Health care utilisation and costs						
*Health care utilisation*	Self-developed questionnaire based on iMTA Medical Consumption Questionnaire [61].	9	N/A	X	X	X
	Client records	N/A	N/A	Continuous registration		

^a^underlined scores indicate the most favourable scores; *N/A* not applicable

#### Clinical outcomes

The primary outcome measure for the CEA will be sedentary time. The primary outcome measure for the CUA will be generic quality adjusted life years (QALYs), measured by means of the standard newest Dutch version of the EuroQol-5D-5 L. (EQ-5D-5 L, [56, 57]). The EQ-5D-5 L consists of five dimensions of health-related quality of life, namely mobility, self-care, daily activities, pain/discomfort and depression/anxiety. Each dimension can be rated at five levels: ranging from ‘no problems’ to ‘major problems’ [58, 59]. The five dimensions can be summed into a health state. Utility values can be calculated for these health states, using preferences elicited from a general population, the so-called algorithm [60]. The utilities at the three time points (baseline and 6 and 12 months after baseline) will be used to calculate QALYs by means of the area under the curve method. In addition, the EQ visual analogue scale will be used to assess current health status [58, 59].

#### Health care utilisation and costs

Volumes of health care utilisation will be measured using a self-developed questionnaire (9 items), which is based on the iMTA Medical Consumption Questionnaire [61]. Additionally, data from client records will be extracted at the end of the study. Overall, the following health care and patient and family costs will be taken into account: 1) primary care (i.e. general practitioner, physiotherapy, day care; 2) hospital care, (i.e. acute care, outpatient medical services and hospital admission; 3) long-term care (i.e. rehabilitation clinic, nursing home and retirement home); 4) home care (i.e. domestic services, personal care, and nursing care); and 5) informal care. Intervention costs will be based on the time health care professionals spent on ‘Stay Active at Home’ training activities. The valuation of health care costs and patient and family costs will be based on the updated Dutch manual for cost analysis in health care research [55]. This manual recommends using standardised cost prices. Cost prices will be expressed in 2017 euros. If necessary, existing cost-prices will be updated to 2017 using the consumer price index.

### Process evaluation

To assess the feasibility of the ´Stay Active at Home´ programme, data from older adults, home care professionals, and other stakeholders (e.g. interventionists, managers) will be collected. A process evaluation plan is designed according to the guidelines of the MRC framework [62]. According to the guidelines key elements are: 1) the implementation of the intervention; 2) its mechanisms of impact; and 3) contextual factors that may affect its implementation and outcomes.

#### Implementation: What is implemented and how?

An intervention may have limited effects either because of weaknesses in its design or because it is not well implemented [62]. To be able to draw reliable conclusions about the effectiveness of the ‘Stay Active at Home’ programme, the implementation of the programme will be evaluated. More specifically, data on treatment fidelity (quality of implementation), dose (quantity of implementation), adaptations (alterations made) and reach (whether the intended audience comes into contact with the intervention) will be collected.

#### Mechanisms of impact: How does the delivered intervention produce change?

For an understanding of how potential effects occur, it is essential to get insight into how an intervention produces change [62]. The ‘Stay Active at Home’ programme aims to change the behaviour of home care professionals from doing things *for* the client towards doing things *with* them. Therefore, the programme intends to 1) increase knowledge; 2) improve self-efficacy and outcome expectations; 3) teach new skills; and 4) provide social support. The process evaluation will examine whether the ‘Stay Active at Home’ programme produces changes through these mechanisms.

#### Context: How does context affect implementation and outcomes?

The implementation and effectiveness of interventions may vary from one context to another due to external factors, which may act as a barrier or a facilitator [62]. Therefore, data from various stakeholders will be collected to get insights into their experiences with the ‘Stay Active at Home’ programme. More specifically, stakeholders will be asked which factors have hindered or facilitated the implementation of Reablement in practice. Insight into these factors is critical to understand the implementation and effectiveness of the ‘Stay Active at Home’ programme.

A mixed-methods design will be chosen for data collection, combining quantitative and qualitative data collection methods. More specifically semi-structured (group) interviews, telephone interviews, a project logbook, registration forms and checklists, client records, and self-report questionnaires will be used to measure the key components. An overview of all data that will be collected according to these three elements is provided in Table 3.

**Table 3:** Overview of process evaluation data collection

Component and definition	Data source	Arm	Data collection method	Specific data^a^	Timing
Implementation					
*Fidelity* Quality of what is delivered	Professionals and other stakeholders (e.g. interventionists, managers)	IG	Semi-structured (group) interviews	– a.o. experienced benefits, burden, usefulness of ‘Stay Active at Home’; involvement with intervention	At the end of the implementation phase
	Older adults	IG, CG	Telephone interviews	– a.o. satisfaction with home care and awareness of behavioural change in professionals	6 months after baseline (with data for effect and economic evaluation)
	Researchers	IG	Project logbook	– Performance according to protocol	Continuously throughout the implementation phase
*Dose* Quantity of what is delivered	Professionals	IG	Registration forms and checklists	– Number of professionals: attending programme meetings; making practical assignments; reading weekly newsletters	Continuously throughout the implementation phase
	Older adults	IG	Client records	– a.o. hours of care; staff turn-over trained professionals; formulation and implementation of goal-setting and action planning	At the end of the implementation phase
*Adaptations* Alterations made to the intervention	Researchers	IG	Project logbook	– if applicable: changes in content, procedures, activities and processes	Continuously throughout the implementation phase
*Reach* Extent to which the target group was reached	Professionals	IG	Project logbook	– Number of professionals who will refuse, drop out or complet the programme and reasons for refusal and drop-out	Continuously throughout the implementation phase
	Older adults	IG	Project logbook	– Number of older adults who will refuse, drop out or complete the programme and reasons for refusal and drop-out	Continuously throughout the implementation phase
Mechanisms of impact					
Mechanisms that are expected to produce change	Professionals	IG, CG	Self-report questionnaire	– Knowledge test and self-efficacy and outcome expectation questionnaire inspired by the work of Resnick et al. [75–77] – Additional evaluation questions (which cover the different topics of the programme)	6 and 12 months after baseline
	Older adults	IG, CG	Self-report questionnaire	– Self-efficacy and outcome expectation questionnaire inspired by the work of Resnick et al. [78]	6 and 12 months after baseline (with data for effect and economic evaluation)
Contextual factors					
Factors that may influence the implementation/ outcomes of the intervention	Professionals and other stakeholders (e.g. interventionists, managers)	IG	Semi-structured (group) interviews	– a.o. facilitators and barriers in applying ‘Stay Active at Home’ in practice	At the end of the implementation phase
	Researchers	IG	Project logbook	– a.o. facilitators and barriers in applying ‘Stay Active at Home’	Continuously throughout the implementation phase

*IG* intervention group, *CG* control group

^a^Need to be further specified

### Sample size

The sample size calculation will be based on the primary outcome of this study, namely sedentary time as measured by the ActiGraph GT9X Link (ActiGraph Inc., Pensacola, FL, USA). The ‘Stay Active at Home’ programme is expected to create a 15% difference in sedentary time (minutes/ day) between the groups. Based on a mean of 535.9 min (*SD* = 145.7) [45] this is equivalent to an effect size of 0.55, which can be interpreted as a medium effect size according to Cohen [63]. To achieve a power of 80% with an alpha of 0.05 (using two-tailed tests) requires a minimum sample size of 54 clients per group *(N =* 108 in total). Considering an expected drop-out rate of 30% before post-test, a total sample size of 154 older adults is needed. Finally, to compensate for a) the inflation of sampling error arising from a clustering effect and b) a mild variation in sample size per nursing team, a correction will be applied, taking into account an intraclass correlation of 0.02 and a coefficient of variation of 0.50, resulting in a total sample size of 260 older adults (130 for each arm) [64].

### Data management

Data are handled confidentially and results will presented in an anonymised way. All original study forms will be entered electronically in Excel 2016 and kept on file at Maastricht University. Forms are stored in numerical order and in a secure and accessible place and manner for a period of 10 years after completion of the study. All records that contain names or other personal identifiers, such as informed consent forms, will be stored separately from study records identified by code number. All local databases will be secured with password-protected access systems. Forms, lists, logbooks, appointment books, and any other listings that link participant ID numbers to other identifying information will be stored in a separate, locked file in an area with limited access. Only two of the involved researchers (authors SFM, THR) will have access to the complete final dataset. Data integrity will be enforced through a variety of mechanisms (i.e. double data entry, range checks for data values). Data will be coded using digital codebooks, which are created for each questionnaire or registration form prior to the start of the study. The option to choose a value from a list of valid codes and a description of what each code means will be available where applicable.

### Data analyses

Missing item responses within a given scale will be replaced by mean imputation [65] using the mean of that client on the other items in that scale at that time point of measurement, assuming that the number of missing item responses does not exceed the missingness percentage suggested by the developers of the given scale. If this information is not available, a missingness percentage of 25% is accepted.

#### Effect evaluation

Descriptive statistics will be used to describe the study groups regarding their sociodemographic characteristics and baseline scores of the primary and secondary outcomes. The primary and secondary outcomes will be analysed according to the intention to treat principle, that is, all available data from all participants will be included in the analysis. Mixed (multilevel) linear regression will be applied with repeated outcome measures (baseline, post-test) nested in clients nested in nursing teams. ‘Nursing team’ is treated as random effect and outcome predictors are: intervention (yes/ no), time (baseline/ post-test), the intervention by time interaction, working area and its interaction with time, as well as the following covariates and their interactions with treatment and time: 1) older adults’ initial level of disability (measured by means of the GARS [50]); 2) client type (existing vs. new clients); 3) working areas. The design, with one team per working area per treatment condition, does not allow including a random team effect and working area by treatment interaction at the same time, because the team effect is completely confounded with the latter interaction. Team is here the unit of randomisation and therefore treated as random. However, to explore interaction of treatment with working area, an additional analysis will replace the random team effect with that interaction and three-way interaction area*treatment*time. If interaction is found, the treatment effect will be evaluated per working area. The software package SPSS for Windows, version 24.0., will be used for all statistical analyses. The level of statistical significance will be set at 0.05 (using two-tailed tests). If interaction effects for the three covariates (i.e. older adults’ initial level of disability; client type; and working area) are present subgroup analyses will be conducted with a significance level of 0.10. The subgroup analyses will have an exploratory purpose only in view of the risk of type I errors due to multiple testing and of type II errors due to reduced sample size.

#### Economic evaluation

For the CEA and CUA incremental cost-effectiveness ratios (ICERs) will be calculated, representing the differences in mean costs between the intervention and control group in the numerator and the difference in mean outcomes in the denominator. Sampling uncertainty around the ICER will be assessed by means of non-parametric boot-strapping (percentile method) [66]. The bootstrapped cost-effectiveness ratios will be subsequently plotted in a cost-effectiveness plane, in which the vertical line reflects the difference in costs and the horizontal line reflects the difference in effectiveness. The choice of treatment depends on the maximum amount of money that society is prepared to pay for a gain in effectiveness, which is called the ceiling ratio. Therefore, the bootstrapped ICERs will also be depicted in a cost-effectiveness acceptability curve showing the probability that ‘Stay Active at Home’ is cost-effective using a range of ceiling ratios. Additionally, to assess the robustness of the assumptions, multi-way sensitivity analyses will be performed. In the sensitivity analysis uncertain factors of assumptions in the base case analysis will recalculated to assess whether the assumptions have influenced the ICERs, for example by varying cost-prices and volumes between minimum and maximum.

#### Process evaluation

For the process evaluation, a combination of quantitative and qualitative data analysis techniques will be used (need to be further specified).

### Research participation: ‘Nothing about us without us…’

To ensure a good match with the target group of the ‘Stay Active at Home’ programme, the experience of relevant stakeholders (i.e. home care professionals, older adults and informal caregivers) have been and will be incorporated in all research phases from pilot work until dissemination/implementation. By incorporating their experiential knowledge in research activities, findings are more likely to be relevant and the likelihood of successful implementation increases [67–69]. In addition, the project is embedded in the Living Lab of Ageing and Long-term Care www.academischewerkplaatsouderenzorg.nl), in which researchers and professionals from various disciplines closely collaborate to develop and disseminate evidence-based healthcare programmes [70].

Arnstein [71] differentiates between eight types of participation, which can be broadly categorised into 1) non-participation (i.e. therapy, manipulation); 2) tokenism (i.e. placation, consultation, informing); and 3) citizen power (i.e. citizen control, delegated power, partnership); Within this study, relevant stakeholders will be involved on different levels, depending on the phase of the project and the wishes of how the stakeholders want to be involved.

#### Informing

We will inform home care professionals and older adults during all phases of the research by making use of newsletters, articles, presentations and symposia. Furthermore, articles will be published in the journals of the involved health care organisation.

#### Consultation

The ‘Stay Active at Home’ programme is developed in close collaboration with relevant Dutch stakeholders (i.e. health care professionals, policy makers, managers, scientists) and a panel of older adults to ensure that all interests are considered and respected in the development [18]. Furthermore, first data about the feasibility of the ‘Stay Active at Home’ programme was collected during an exploratory trial (ClinicalTrials.gov: NCT02904889 (Smeets RGM, Kempen GIJM, Hansen WAG, Zijlstra GAR, van Rossum E, de Man-van Ginkel JM, et al. Experiences of home care professionals with the Stay Active at Home programme targeting reablement of community-living older adults. A qualitative study, submitted for publication)). During the proposed study, additional data from home care professionals and older adults will be collected as part of the process evaluation.

#### Placation

During the study two authors (SFM and THR) will have continuously contact with different stakeholders from MeanderGroep South-Limburg (i.e. training officers, managers of nursing teams/domestic teams and district nurses) to make sure that the training fits with their working routines and to exchange experiences about the progress. In addition, a steering group will be created consisting of at least one representative from all collaborators (i.e. Maastricht University, Zuyd University of Applied Sciences, MeanderGroep South-Limburg, GP association in South Limburg (OZL General Practitioners), Burgerkracht, Dutch Nursing Association (V&VN)) and the research partners (also see the next paragraph). The steering group will meet twice a year to discuss the progress of the study and the dissemination/implementation of the results.

#### Partnership

During the full trial period, four research partners (i.e. one nurse, one DSW, one older adult and one informal caregiver) will be extensively involved in the research activities. Together with author THR they will prepare and execute the research activities and will disseminate/implement the results. For example, they are involved in the preparation and execution of qualitative data collection and analysis, writing articles and giving presentations. The representatives of older adults and informal caregivers are supported by an employee of the Burgerkracht (author MV), who will meet regularly with them and the authors SFM and THR to talk about the process of involvement. They can contact MV if they need support in their role to participate in the project.

### Trial status

The recruitment of home care professionals started in September 2017. The first older adults were enrolled in October 2017. At the same time the data collection of the baseline data started. The last older adults will be recruited by the end of June 2018. Consequently, the last follow-up measurements will be conducted in June 2019. First results are expected by the end of 2019.

## Discussion

‘Stay Active at Home’ is a training programme that aims to equip home care professionals with knowledge, self-efficacy, skills and social support to deliver day-to-day services at home according to the principles of Reablement. This two-group cluster randomised controlled trial will be conducted to evaluate whether its implementation leads to a reduction in sedentary behaviour in older adults and thereby an increase in their level of physical activity. Furthermore, it will be investigated whether the programme leads to more cost-effective outcomes in terms of older adults’ health and wellbeing. In addition, an extensive process evaluation will be conducted alongside the trial. The process evaluation is of utmost importance to explain the results of the effect and economic evaluation.

This study has several strengths. First, the ‘Stay Active at Home’ programme was developed based on international evidence and in close collaboration with Dutch and foreign stakeholders [18]. Second, prior to the present study an exploratory trial was conducted (ClinicalTrials.gov: NCT02904889 (Smeets RGM, Kempen GIJM, Hansen WAG, Zijlstra GAR, van Rossum E, de Man-van Ginkel JM, et al. Experiences of home care professionals with the Stay Active at Home programme targeting reablement of community-living older adults. A qualitative study, submitted for publication)). The aims of this exploratory trial were to obtain experiences with the ‘Stay Active at Home’ programme and to test the study design. During the exploratory trial, challenges were identified which led towards some adaptations regarding the programme and the study design. For example, the exploratory trial showed that home care professionals did not identify with the interventionists, which is essential requirement for successful behavioural change according to behaviour change theory [72, 73]. Therefore, professionals who have already followed the ‘Stay Active at Home’ programme will be used as role models during the upcoming training sessions to share their experiences with their colleagues. The recruitment procedure was also adapted. In the exploratory trial, the participating nursing teams were asked to recruit older adults, resulting in a low response rate as nurses felt not responsible for the recruitment. In the proposed cluster randomised controlled trial, older adults will receive a short informational letter and flyer about the study as an announcement for a subsequent telephone call to assess whether they are potentially interested in participating in this study. Written informed consent will be obtained by the research team during the first home visit. Third, a strong aspect of the current study is that a combination of effect, economic and process evaluations will be conducted. Randomised controlled trials in the field of Reablement combining these different evaluations are scarce, yet important to obtain a complete picture. However, some limitations of this study must be acknowledged. First, it is not feasible in this study to objectively measure whether a behavioural change in home care professionals has taken place, as it is not possible to make use of (video) observations in the home care setting. Therefore, we rely on self-reported behaviour by home care professionals, which can result in biases due to social desirability and unaware/unskilled behaviour. Second, for this study, a follow-up period of 12 months has been chosen. This period may be too short to show effects, as home care professionals must first change their own behaviour before we can expect a behavioural change in older adults or changes in the consequent cost-effective outcomes with regard to their health and wellbeing. However, a longer follow-up is not possible due to practical and financial reasons.

## Abbreviations

ADL: Activities of daily living
CEA: Cost-effectiveness analysis
CG: Control group
CUA: Cost-utility analysis
DSM-IV: Diagnostic and Statistical Manual of Mental Disorders, 4th Edition
DSWs: Domestic support workers
EQ-5D-5 L: EuroQol-5D-5 L
GARS: Groningen Activity Restriction Scale
Hz: Hertz
IADL: Instrumental activities of daily living
ICERs: Incremental cost-effectiveness ratios
IG: Intervention group
iMTA: Institute for Medical Technology Assessment
LASA: Longitudinal Aging Study Amsterdam
MRC: Medical Research Council
N/A: Not applicable
PHQ-9: Patient Health Questionnaire 9
QALYs: Quality adjusted life years
SD: Standard deviation
SPIRIT: Standard Protocol Items: Recommendations for Interventional Trials
SPPB: Short Physical Performance Battery
SPSS: Statistical Package for the Social Sciences
T0: Baseline
T1: Follow-up 1
T2: Follow-up 2
UK: United Kingdom
US: United States
